# Membrane dynamics and organelle biogenesis—lipid pipelines and vesicular carriers

**DOI:** 10.1186/s12915-017-0432-0

**Published:** 2017-10-31

**Authors:** Christopher J. Stefan, William S. Trimble, Sergio Grinstein, Guillaume Drin, Karin Reinisch, Pietro De Camilli, Sarah Cohen, Alex M. Valm, Jennifer Lippincott-Schwartz, Tim P. Levine, David B. Iaea, Frederick R. Maxfield, Clare E. Futter, Emily R. Eden, Delphine Judith, Alexander R. van Vliet, Patrizia Agostinis, Sharon A. Tooze, Ayumu Sugiura, Heidi M. McBride

**Affiliations:** 1 MRC Laboratory for Molecular Cell Biology, University College London, Gower Street, London, WC1E 6BT UK; 2 Cell Biology Program, The Hospital for Sick Children and Department of Biochemistry, University of Toronto, Toronto, Canada; 3 Université Côte d’Azur, CNRS, Institut de Pharmacologie Moléculaire et Cellulaire, Valbonne, France; 4 Department of Cell Biology, Yale University School of Medicine, New Haven, CT 06520 USA; 5 Department of Neuroscience and Cell Biology, Howard Hughes Medical Institute, Kavli Institute for Neuroscience and Program in Cellular Neuroscience, Neurodegeneration, and Repair, Yale University School of Medicine, New Haven, CT 06510 USA; 6 NICHD, NIH, Bethesda, MD USA; 7 HHMI Janelia Research Campus, Ashburn, VA USA; 8 UCL Institute of Ophthalmology, 11-43 Bath Street, London, EC1V 9EL UK; 9 Genentech, 1 DNA Way, South San Francisco, CA 94080 USA; 10 Department of Biochemistry, Weill Cornell Medical College, 1300 York Ave, New York, NY 10065 USA; 11 Molecular Cell Biology of Autophagy Laboratory, The Francis Crick Institute, London, UK; 12 Laboratory of Cell Death Research and Therapy, Department of Cellular and Molecular Medicine, KU Leuven, Leuven, Belgium; 13 Kobe University Graduate School of Medicine, 1-5-6 Minatojima-minamimachi, Chuo-ku, Kobe, Hyogo 650-0047 Japan; 14 Montreal Neurological Institute, McGill University, 3801 University Avenue, Montreal, Quebec H3A 2B4 Canada

## Abstract

Discoveries spanning several decades have pointed to vital membrane lipid trafficking pathways involving both vesicular and non-vesicular carriers. But the relative contributions for distinct membrane delivery pathways in cell growth and organelle biogenesis continue to be a puzzle. This is because lipids flow from many sources and across many paths via transport vesicles, non-vesicular transfer proteins, and dynamic interactions between organelles at membrane contact sites. This forum presents our latest understanding, appreciation, and queries regarding the lipid transport mechanisms necessary to drive membrane expansion during organelle biogenesis and cell growth.

## Tapping into the routes for membrane expansion

### Christopher J. Stefan

Plasma membrane expansion is intrinsic to balanced cell growth and cell size control. Cellular volume and surface area adjust to accommodate newly synthesized and acquired materials. Consequently, metabolism becomes detrimental if cell-surface growth is compromised. A requirement for coordinated membrane lipid and cytoplasmic macromolecular biosynthesis is highlighted by seminal studies describing “inositol-less death” in yeast cells. Upon disruptions in phosphatidylinositol lipid synthesis, cell-surface expansion terminates while cytosolic constituents continue to accumulate [1]. This imbalance in cell volume and cell density control leads to increased internal turgor pressure and eventually cell rupture. Cellular integrity not only requires bulk plasma membrane (PM) growth, but precise regulatory control of PM lipid content as well. The PM has a unique lipid composition that is enriched in certain sterol, sphingo-, and phospholipids compared to other cellular membranes [2]. This PM lipid identity is conserved across eukaryotic cells and is critical for PM organization and integrity. But how is the distinct composition of the PM achieved and what are the lipid delivery mechanisms necessary for PM biogenesis and homeostasis?

**Fig. 1.**
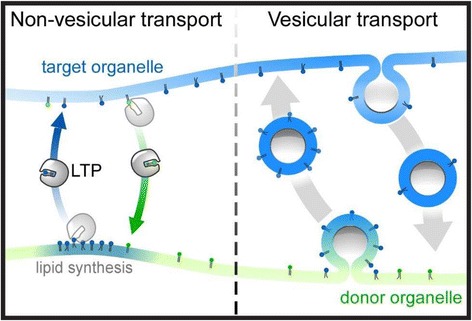
Membrane lipid flow occurs via transport vesicles and non-vesicular transfer proteins at membrane contact sites. But how does the cell use these essential delivery routes, as needed, for membrane expansion and organelle biogenesis? Moreover, how is membrane lipid composition precisely regulated to maintain organelle homeostasis?

One vital pathway is vesicular membrane trafficking. Notably, mutants defective in the yeast secretory pathway were originally isolated based on their increased density, as protein synthesis continues whereas cell-surface growth ceases upon intracellular accumulation of PM-bound vesicles [3]. The similarities between inositol-starved and secretory defective yeast cells indicate that vesicular intermediates, at least in part, couple lipid biosynthesis to PM delivery. Of significance, however, while PM expansion is impaired in secretory mutant cells, phospholipid biosynthesis is not interrupted [4]. This is likely because membrane lipids not only traverse the secretory pathway but also have additional routes for their distribution throughout the cell. This includes both vesicular trafficking and non-vesicular transport between cellular organelles (Fig. 1).

Non-vesicular delivery of lipids became evident upon the discovery that cholesterol and secretory cargo proteins synthesized in the endoplasmic reticulum (ER) undergo distinct rates of transport to the PM [5]. Further work by the Simoni and Pagano laboratories found that phospholipids are also rapidly transported to the PM. More recent studies have provided key mechanistic insight into this process. A conserved family of lipid transfer proteins, the oxysterol-binding protein related proteins (ORP), has been demonstrated to transfer newly synthesized lipids including cholesterol and phosphatidylserine from the ER in exchange for the phosphoinositide isoform phosphatidylinositol 4-phosphate (PI4P) generated at target organelles [6–11]. As such, cells utilize PI4P metabolism for the transport of lipids (see accompanying section by G. Drin). Phosphoinositide lipids are thought to be rare membrane components, and one question is whether there is sufficient PI4P synthesis to drive all sterol and phosphatidylserine transport to the PM. However, PI4P is the most abundant phosphoinositide species in eukaryotic cells, consistent with a role in bulk lipid transport. In yeast, phosphatidylserine constitutes approximately 2% (mol) of cellular lipids while phosphatidylinositol, used to generate PI4P, makes up 20% of cellular lipids [12]. PI4P levels are generally 1% of phosphatidylinositol [13], and thus PI4P steady state levels appear to be only 0.2% of total cellular lipids. However, PI4P is continuously synthesized and turned over, and PI4P levels increase by an order of magnitude upon loss of PI4P phosphatases [13]. Thus, PI4P may make up 2% of cellular lipids, similar to levels of phosphatidylserine in the cell. PI4P exchange could therefore extensively drive the enrichment of phosphatidylserine at the PM.

However, is PI4P exchange the major mechanism for lipid delivery to the PM? ORP family members have also been implicated in the transport of sterol lipids [7, 9]. Sterol lipids constitute approximately 14% (mol) of total cellular lipids in yeast [12], suggesting that sterol and phosphatidylserine significantly outnumber PI4P in the cell. Possibly, sterol lipids and phosphatidylserine have longer lifetimes, resulting in apparently large differences in steady-state levels. Ceramides are also transferred from the ER to the late Golgi network by the PI4P-regulated lipid transfer protein CERT [14]. While the extent of PI4P-mediated lipid transfer from the ER is not entirely clear, PI4P metabolism may be greatly underestimated and even match lipid transfer rates, as needed. Consistent with this, phosphatidylserine synthesis in the ER decreases upon inhibition of PI4P metabolism [11]. Some of this load may be handled by additional transfer mechanisms, including the steroidogenic acute regulatory protein (StAR)-related lipid-transfer (StART) domain family members that are major sterol carriers in the cell (see accompanying section by Iaea and Maxfield). In addition, certain lipids, such as ceramides, are selectively packaged into vesicular carriers for ER export [15]. Importantly, disruption of PI4P metabolism is lethal and PI4P is required for trafficking along the early secretory pathway [13]. At late Golgi compartments, PI4P further controls the sorting of membrane proteins into sterol- and sphingolipid-enriched vesicles destined for trafficking to the PM [16], and PI4P itself exits the late Golgi network on secretory vesicles. Accordingly, lipids are delivered to the PM in steps that involve both non-vesicular and vesicular mechanisms, and PI4P regulates both of these processes.

What then are the relative contributions of vesicle trafficking and non-vesicular transport mechanisms in the delivery of lipids to the PM? This question remains a challenge to disentangle experimentally in part because lipids utilize multiple routes that may compensate for one another. One example is the trafficking of phosphatidylserine throughout the cell. Loss of the ORP isoforms implicated in transfer of phosphatidylserine from the ER to the PM in yeast does not eliminate delivery of phosphatidylserine to the PM and does not impair cell growth [8, 10]. Previous work has suggested that phosphatidylserine is delivered to the PM at sites of polarized growth via secretory vesicles, as a phosphatidylserine reporter was observed on vesicles in secretory mutants [17]. Thus, lipids enriched at the PM may be delivered to the PM by non-vesicular transfer from the ER and by vesicular carriers that transport both lipids and proteins along the secretory pathway.

Lipid transfer proteins function at organelle contacts—where close apposition of the donor and acceptor membranes would facilitate lipid transfer [2] (see accompanying section by T. Levine). One surprising observation, however, is that several of the proteins that form and function at ER–PM contacts, including the ER-localized VAP and E-Syt family members, are not essential for cell growth [18, 19]. Lipid transfer proteins, including the ORPs, may function outside the context of an ER–PM contact. However, PI4P accumulates at the PM in yeast cells lacking the VAP and E-Syt orthologs, indicating that ORP-mediated lipid exchange between the ER and PM is compromised [19]. This suggests that other membrane lipid transfer mechanisms compensate for impaired lipid transport activities at ER–PM contacts. Possibly, PI4P-mediated lipid transfer between the ER and Golgi network is sufficient for cell viability and growth. Upon loss of ER–PM contacts, lipids that normally traffic directly from the ER to the PM may be re-routed to Golgi compartments and then packaged into vesicular carriers bound for the PM. While speculative, this suggests that loss of specific lipid transfer activities may be bypassed by flux into other trafficking pathways.

Although compensatory cross-talk between non-vesicular and vesicle trafficking pathways remains tentative, it is clear that polarized secretion is necessary for PM expansion. As mentioned, yeast cells lacking an intact secretory pathway fail to grow and increase in size [3]. Moreover, in budding yeast, the ER is inherited into a daughter cell that has already formed [20]. This suggests that polarized secretion initially drives PM expansion at sites of growth. Interestingly, yeast cells depleted of the VAP proteins have an aberrant cell morphology resembling hyperpolarized growth [19, 21]. Thus, ER inheritance and formation of an extensive cortical ER–PM contact network may mark a switch in modes for PM expansion, from polarized growth driven by targeted secretory vesicles to isotropic growth facilitated by non-vesicular lipid delivery from the ER to the PM. Non-vesicular lipid transport may also be critical for maintaining the unique lipid composition of the PM. Similar to ER inheritance in yeast, myosin motors transport the ER along actin cables into newly formed dendritic spines in neurons [22]. It is not yet clear if or how the ER is involved in dendritic spine growth and shape control. Cells depleted of the VAPs and additional proteins proposed to function at ER–PM contacts display neurite outgrowth defects [23]. Yet loss of the VAP proteins impairs both non-vesicular lipid transport and vesicular trafficking [9, 24]. Consistent with this, VAPs are implicated in directed transport of endosomes along microtubules during neurite outgrowth, further supporting a role for vesicular trafficking in PM expansion [25].

Membrane lipid transfer occurs in the absence of vesicular trafficking and non-vesicular mechanisms can account for rapid bulk lipid flow [2]. However, we must continue to examine the interplay between non-vesicular and vesicular pathways and how they may act in concert for directional net movements of lipids. In addition, rapid membrane expansion is crucial for organelle biogenesis and dynamics, including phagosomes, autophagosomes, the Golgi network, endosomes, mitochondria, and peroxisomes—topics further discussed in this forum. Future studies on cross-talk between membrane lipid delivery pathways will certainly have tremendous impact on our understanding of the mechanisms for membrane expansion and cell size and growth control.

## Membrane expansion in the course of phagocytosis

### William S. Trimble and Sergio Grinstein

Phagocytosis—the engulfment of particulate material—is an ancient process, developed by protozoans to ingest nutrients. In metazoans phagocytosis of pathogens plays a crucial role in innate immunity, and the clearance of apoptotic cells is key to tissue homeostasis and remodelling. Phagocytosis culminates with the closure and scission of a plasma membrane-derived vacuole, the phagosome, which proceeds to mature, becoming an effective microbicidal and degradative compartment. The size of phagosomes is dictated by the size of the target particle; engulfment of apoptotic cells or of fungal hyphae requires the formation of very large vacuoles.

Internalization of a large area of plasmalemma would inevitably be expected to reduce the surface of the phagocytic cell, eventually limiting further ingestion. Remarkably, however, neutrophils and macrophages can engulf multiple large particles. Indeed, internalization of particles larger than the phagocytic cell itself is not uncommon. How is this feat accomplished?

A clue to the underlying mechanism was initially provided by electrophysiological experiments; capacitance measurements revealed that the cell surface area of macrophages does not decrease in the course of phagocytosis, but can actually *increase* [26]. This conclusion was subsequently validated using solvochromic fluorescent dyes in cells performing frustrated phagocytosis of large, flat surfaces [27].

The simplest way to account for the preservation of the surface area in the face of ongoing internalization is to postulate the occurrence of concomitant exocytosis. This notion was initially tested and validated measuring the displacement towards the cell periphery of endomembrane vesicles. The exposure on the cell surface of epitopes originally located in the lumen of endomembrane vesicles confirmed the occurrence of exocytic fusion [28]. Remarkably, these experiments also revealed that the compensatory exocytic events do not occur randomly, but are largely restricted to the site where the particle is being ingested.

The nature of the endocytic compartment delivered to the surface in response to phagocytic stimuli has been the source of debate; recycling endosomes [28], late endosomes [29] and even lysosomes [30] have been invoked as contributors. It is conceivable that the type and number of compartments mobilized varies with the phagocytic signal. In this regard, it is noteworthy that phagocytosis can be initiated by a variety of opsonic and non-opsonic receptors, and that even when a single, defined receptor type is engaged, the nature and intensity of the signaling cascade elicited depends on the size of the targets and the density of ligands on their surface. The most striking example is provided by phosphatidylinositol 3-kinase, which is absolutely essential for the ingestion of large (≥5 μm) particles, yet is dispensable for small ones [27, 31].

The molecular machinery driving exocytosis during phagosome formation has not been characterized in sufficient detail. While it is clear that membrane fusion is mediated by SNAREs, including SNAP23, the motor(s) driving the vesicles towards the base of the nascent phagosomes, the signals initiating this displacement and the triggers of the membrane fusion step remain obscure. Unlike other types of stimulated exocytosis, cytosolic calcium changes are seemingly not required in (at least some types of) phagocytosis. The localized disappearance of phosphatidylinositol 4,5-*bis*phosphate [32] appears to be the common, *sine qua non*, event reported in all instances; whether it is the disappearance of the phosphoinositide itself and/or the concomitant generation of metabolites such as diacylglycerol or phosphatidic acid is also unclear.

In summary, phagocytosis provides a prototypical example of membrane expansion. Expansion occurs acutely, locally and on demand, and serves not only to maintain surface membrane homeostasis, but also to secrete cytokines [33] and to initiate the process of phagosomal maturation, thereby expediting the killing of pathogens and the digestion of dead cells and debris.

## PI4P to synchronize lipid transport with vesicular trafficking

### Guillaume Drin

A remarkable feature of eukaryotic cells is the abundance of a special lipid, the sterol, in their outermost membrane, the plasma membrane (PM), relative to internal organelles. Sterol represents only 1–5% of lipids in the endoplasmic reticulum (ER), more in the Golgi complex and up to 40% in the PM [34]. Such an accumulation is critical. Due to its rigid structure, sterol reduces the flexibility of neighboring lipids, making the PM both thick and impermeable. Yet most of sterol originates from the ER, as it is synthesized there or destocked from lipid droplets. Thereby, it seemed obvious that mechanisms dedicated to transport sterol from the ER to PM come into play in generating the sterol gradient observed in the cell. Early studies demonstrated that sterol does indeed move from the ER to PM, but independently of transport vesicles that circulate throughout the secretory pathway. Notably, in yeast, shutting down almost all vesicular routes by silencing a key protein, Sec18, was found to have no impact on direct ER-to-PM sterol transfer [35]. It was thus proposed that sterol is mainly conveyed along non-vesicular routes by lipid transfer proteins (LTPs) able to help this very hydrophobic molecule to cross the water ‘wall’ between organelles.

Some of us wished to better describe how this transport occurs. An interesting model came from observations showing that a lack of sphingolipids, a class of lipid almost exclusively found in the PM, precludes sterol accumulation. Sterol has a preferential affinity for sphingolipids and is presumably trapped in the PM at the expense of the ER. It was thus proposed, with no further investigations, that LTPs able to shuttle sterol randomly between the ER and the PM, backed by the thermodynamic trap in the PM, create a sterol gradient between the two membranes. For our part, we shed new light on Osh4/Kes1, a protein of the Osh/ORP family, suggested to be a sterol transporter in yeast. We found something intriguing: Osh4 can exchange sterol with a second lipid called phosphatidylinositol 4-phosphate (PI4P) [7]. PI4P is made in an energy-dependent manner at the trans-Golgi and PM, and is prominent in these regions, whereas a hydrolysis reaction prevents any accumulation in the ER. This led to an appealing idea: this imbalance of PI4P might be used by Osh4 to transport sterol from the ER to the trans-Golgi or PM. In one cycle, moving through the cytosol by diffusion, Osh4 would extract a sterol molecule from the ER, exchange it with PI4P at the Golgi membrane, and then deposit PI4P at the ER. In vitro, Osh4 transports sterol in a vectorial manner between two membranes, by dissipating a PI4P gradient, and can create a sterol gradient in return [36]. Thus, Osh4 seems to be a perfect molecular device to exploit PI4P turnover for creating a sterol gradient in cells. Yet a question we face is whether or not the transport of sterol measured in vitro is really happening in cells. Indeed none of the seven Osh proteins seems to be a LTP able to ensure the large sterol fluxes measured at the ER–PM interface in yeast [37]. More recently we have learned from structural and functional analysis that many of them are unable to bind sterol (reviewed in [38]). Regarding Osh4, the debate is further complicated by evidence of its regulatory role in polarized exocytosis [39], a role that seems at first glance difficult to link with a lipid transport function.

**Fig. 2.**
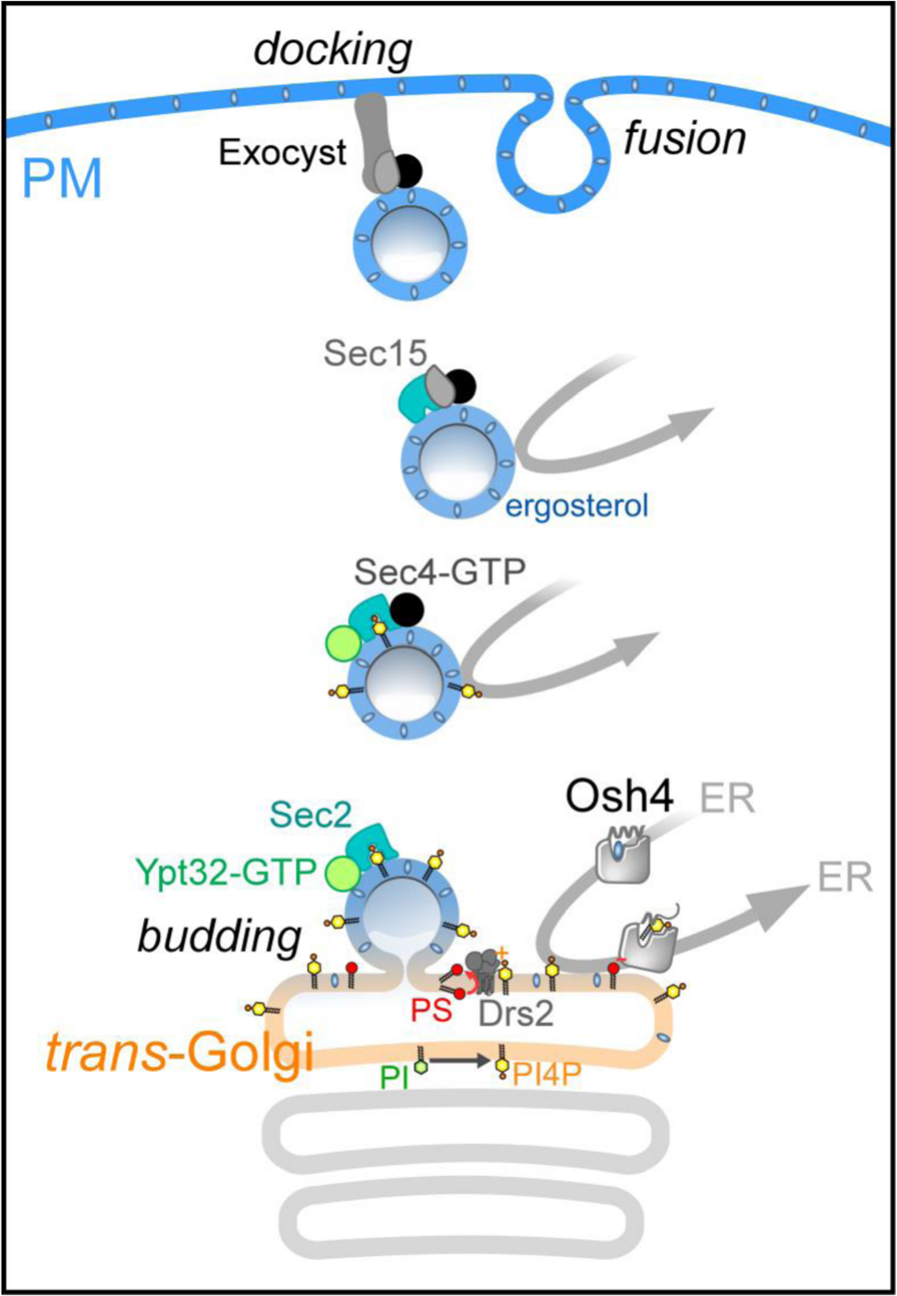
The budding of exocytic vesicles presumably depends on the association of sterol with sphingolipids into microdomains and the flip-flop of PS catalyzed by Drs2. By sterol/PI4P exchange, Osh4 would supply the trans-Golgi with sterol (made in the ER) while removing PI4P (then transported back to the ER and hydrolyzed). Sterol import might be coordinated with the flippase activity of Drs2, repressed by Osh4 via PI4P removal. The exposition of PS on the cytosolic side of the trans-Golgi can in turn downregulate Osh4. Once released, vesicles dock to the PM prior to fusion. Ypt32, a Rab protein that promotes vesicle formation, also initiates a cascade of events leading to the docking process. The GTP-bound form of Ypt32 associated with PI4P recruits Sec2. Then Sec2 activates the Rab Sec4p that in turn recruits Sec15, an exocyst subunit. Osh4 extracts PI4P and the lack of PI4P triggers a conformational change in Sec2 that gains avidity for Sec15. The formation of Sec2-Sec4-Sec15 complexes makes vesicles competent for docking. Simultaneously, due to its exchange activity, Osh4 likely reinforces the enrichment of vesicles in sterol. All these elements suggest that Osh4 synchronizes the build-up of sterol in vesicles with key steps of their life cycle, thereby ensuring an appropriate lipid composition and organization of the PM during its expansion

Polarized exocytosis relies on vesicles that transport proteins from the trans-Golgi to the PM (Fig. 2). Once detached from the parental compartment, these vesicles move along actin cables, dock to the PM, and deliver their content by fusion. These events are initiated by proteins that bind PI4P on the surface of nascent vesicles, but PI4P must disappear afterward to allow the docking process [40]. The budding process itself is mysterious and would depend on lipid-dependent remodeling processes occurring at the trans-Golgi. A hypothesis is that co-segregation of sterol with sphingolipids into ordered domains is critical for the budding of post-Golgi vesicles. Interestingly, sterol accounts for 10% of lipids in the trans-Golgi and twice more in exocytic vesicles [41], suggesting that these vesicles might be sterol conveyors contributing to the build-up of this lipid in the PM. In fact, many observations suggest that the life cycle of these vesicles directly relies on the lipid exchange activity of Osh4. Indeed, Osh4 reduces the availability of PI4P in the trans-Golgi and lowers cellular PI4P levels [42]. This suggests that Osh4 consumes PI4P to supply the trans-Golgi with sterol, allowing its proper association with sphingolipids and eventually vesicle genesis. Interestingly, Osh4 coordinates with Drs2, a flippase that displaces mainly phosphatidylserine (PS) through the trans-Golgi membrane. The asymmetry that is generated in the membrane also promotes budding processes essential for exocytosis [43]. Osh4 inhibits Drs2, likely by removing PI4P, and in return, the exposition of PS by Drs2 could inhibit sterol delivery by Osh4 [44]. At the post-Golgi level, Osh4 removes PI4P from exocytic vesicles en route to the PM, making them competent for docking [45]. One might thus posit that, prior to the docking step, Osh4 completes the enrichment of newly formed vesicles with sterol via exchange with the last remaining PI4P molecules. Thus, a potent idea is that Osh4 uses its exchange capacity to play a key role during the remodeling of the trans-Golgi membrane and post-Golgi trafficking. This would explain why the absence of Osh4 impacts the sterol distribution in the PM [37].

What comes out of this scenario is that PI4P, both a fuel for lipid exchange by Osh4 and a molecular cue recognized by PI4P-targeting effectors, can synchronize sterol enrichment and hence membrane maturation with other crucial events for exocytosis. Thus, in yeast, PI4P appears clearly as a functional node between non-vesicular lipid transport processes and vesicular trafficking. These data also force us to reconsider that a sterol fraction might be selectively conveyed by transport vesicles to the PM. Given its abundance and lipid exchange rate, Osh4 action coupled to exocytosis could provide up to 60% of sterol required for the expansion of the PM surface during asymmetric division of yeast [36]. A good move would be to analyze the sterol content of secretory vesicles when Osh4 is absent. More generally, it would be great to improve our approaches to track the ER-to-Golgi sterol transport mediated by LTPs and its contribution to the generation of the cellular sterol gradient.

Lipid exchanges fueled by PI4P metabolism increasingly appear as a widespread mechanism in eukaryotes. In human, sterol–PI4P exchange occurs in ER–Golgi contact sites, mediated by OSBP, an Osh4 homologue [9]. OSBP is more complex and needs to be anchored to the trans-Golgi via a PI4P-binding domain for its exchange efficiency. Because it consumes PI4P for sterol transport, OSBP can also regulate its residence time at contact sites and likely the PI4P-binding protein CERT that transports sphingolipid precursors [9]. Here, PI4P might serve to orchestrate the co-enrichment of sterol and sphingolipids in the trans-Golgi directly. Recently, we and others have established that Osh6/Osh7 and their closest homologues in human, ORP5/ORP8, are PS/PI4P exchangers [6, 10]. We now understand that PI4P metabolism drives the accumulation of ER-derived PS in the PM, where it plays a ke*y* role as a molecular signpost and activator of signaling proteins. Defining whether these novel exchange routes are coordinated with other PI4P-dependent mechanisms and associated with membrane remodeling processes should be a matter of exciting research in the future.

## Membrane tethering and lipid transport by SMP domain-containing proteins

### Karin Reinisch and Pietro De Camilli

Contacts between intracellular membranes are well-established key players in a variety of intracellular processes, including regulation of cytosolic calcium levels and control of lipid homeostasis. However, much remains to be learned about the molecular mechanisms operating at these sites. An approach toward a better understanding of these mechanisms is to characterize the protein tethers mediating these contacts, to elucidate how they are localized, how they function, and how they are regulated. One class of such tethers are proteins that contain a TULIP lipid transport module, and more specifically the intracellular version of this domain, called the SMP (*s*ynaptotagmin-like, *m*itochondrial and lipid-binding *p*roteins) domain [46]. Several such proteins have been identified. They include the extended synaptotagmins (E-Syts, known as tricalbins in yeasts), conserved in all eukaryotes, and TMEM24, a more specialized protein present only in metazoans and enriched in cells of neuroendocrine lineage, all of which are targeted to contacts between the endoplasmic reticulum (ER) and the plasma membrane [18, 19, 47]. They also include the conserved protein Nvj2/Tex2, which is localized at contacts between the ER and either the vacuole or the Golgi complex in yeast [48, 49], and three (Mmm1, Mdm12, and Mdm34) of the four subunits of the ER–mitochondrial encounter structure (ERMES) complex, which is localized at ER–mitochondrial contacts in yeasts and other fungi [50], but is not present in metazoans.

**Fig. 3.**
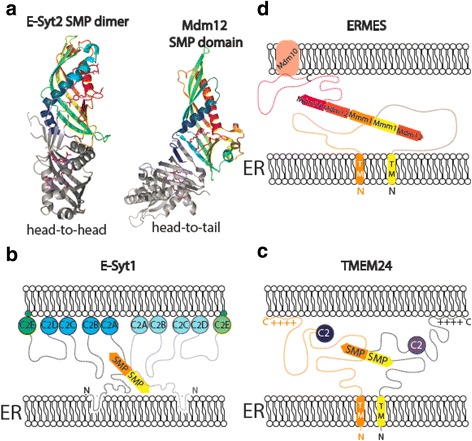
Architecture of SMP-domain containing proteins. **a** *Left*: ribbon diagram of the SMP dimer of E-Syt2 (PDBID 4P42). One SMP domain is colored from *blue* at the N-terminus to *red* at the C-terminus. Lipid and detergent molecules in the hydrophobic channel are shown in *pink. Right*: two Mdm12 monomers arranged head-to-tail as observed in the crystal (PDBID 5GYD). Mdm12 and Mmm1 may interact similarly within ERMES. **b**–**d** Tethering for E-Syt1, TMEM24, and ERMES, as indicated

A crystal structure of a portion of E-Syt2 showed that its SMP domain shares a fold with modules in the TULIP family [51], first identified in extracellular proteins involved in lipid transport outside cells, such as CETP and BPI. It demonstrated that the E-Syt2 SMP module dimerizes in a ‘head’-to-‘head’ manner to form a tube-like structure with a hydrophobic cavity, which runs along its length and is connected to the solvent with a seam also spanning its entire length (Fig. 3a). The crystal structure also showed a mixture of phosphatidylethanolamine and phosphatidylglycerol, which had co-purified with the protein, bound with their acyl chains in the hydrophobic channel and their hydrophilic headgroups extruded through the seam and into solvent, where they were disordered. The SMP domain of TMEM24 dimerizes as well [47], likely in a similar fashion to that of E-Syt2, as does the SMP domain of Mmm1 [52], one of the components of the ERMES complex. In ERMES, additionally, the ‘tail’ end of each Mmm1 dimer associates with the ‘head’ end of the SMP domain from an Mdm12 subunit to form a longer tube-like structure [52]. How the third SMP domain protein in ERMES, Mdm34, associates with the Mmm1/Mdm12 heterotetramer is not known, although it is likely that its SMP module interacts with the heterotetramer in such a way as to lengthen the tubular structure even further.

The details of lipid binding differ among SMP domains, as they do across the entire TULIP domain 7family. For example, two lipid molecules are bound per SMP module in E-Syt2 (four in the dimer) [51], whereas each TMEM24 and Mdm12 SMP module accommodates only one [47, 53]. More importantly, SMP domain proteins differ in their lipid harboring and transport preference, based on biochemical and liposome-based lipid transport assays.

Which lipids the SMP domain proteins transfer in the context of the living cell is a topic under active investigation. The detection by mass spectrometry in the SMP domain of the E-Syts of a variety of glycerophospholipids, but not of lipids in other classes, suggested that they could play a role in bulk transfer of these lipids between the ER and the plasma membrane [51]. Subsequent studies of E-Syt knockout cells revealed a role for these proteins in clearing the accumulation of diacylglycerol in the plasma membrane, most likely by transferring it to the ER, after its acute generation in the plasma membrane in response to PI(4,5)P_2_ hydrolysis [54]. It remains unknown whether diacylglycerol is transported along with other glycerophospholipids during bulk transport or whether it is preferentially transported. For example, it is possible that the bulky headgroups present in other glycerophospholipids reduce their affinity for the E-Syts and hence their transport rate as compared to diacylglycerol, which lacks a headgroup. In previous in vitro assays, lipid transfer by the E-Syts was assessed by FRET using fluorescently tagged cargo lipids [54, 55]. Further studies based on transport assays involving natural lipids versus chemically modified ones should help to resolve this question.

TMEM24 was shown preferentially to bind and transport PI, suggesting a role for this protein in delivery of newly synthesized PI from the ER to the PM to replenish PI(4,5)P_2_ pools depleted during phospholipase C signaling [47]. While ERMES’ SMP domains also transport glycerophospholipids, their preference is for phosphatidylcholine [52]. NVJ2 was shown to impact ceramide transport, thus pointing to this lipid as a cargo of its SMP domain, but there is no direct evidence, so far, that it can bind and transport this lipid [48].

The function of all known SMP domain-containing proteins, or their complexes, in lipid transport is tightly interrelated with their property to tether membranes. Indeed, overexpression of these proteins expands the area of contact sites in the cell [18, 47, 56]. Typically, the SMP is bracketed via unstructured linkers between protein regions that connect two different membranes, so that it can ferry lipid cargo between the two bilayers. The E-Syts are anchored to the ER membrane via an N-terminal ß-hairpin, and bind the plasma membrane via C2 domains (Fig. 3b) [18, 56]. The most C-terminal C2 domain recognizes the phosphoinositide PI(4,5)P_2_, which is specifically enriched at the plasma membrane, and is thus responsible for the selective localization of the E-Syts at ER–plasma membrane contacts sites [18, 54]. Other C2 domains of the E-Syts interact with membranes in response to Ca^2+^, thus regulating tethering [18, 54, 56–58]. TMEM24 is also anchored to the ER via its N-terminal region, but its single C2 domain does not play a major role in plasma membrane binding and its function remains unresolved. Instead, binding of TMEM24 to the plasma membrane is mediated by its highly conserved C-terminal region, which is enriched in basic residues and thus optimally suited to bind the cytosolic leaflet of this membrane, which is highly acidic due to the presence of phosphoinositides and to the high concentrations of phosphatidylserine [47] (Fig. 3c). The ERMES complex is anchored in the ER membrane via the N-terminal transmembrane region of Mmm1 and binds mitochondria via an interaction of Mdm34 with the integral membrane protein Mdm10 in the outer mitochondrial membrane [50, 59] (Fig. 3d). Nvj2 has a predicted N-terminal transmembrane segment anchoring it to the ER, and is thought to bind other membranes in *trans* through a PH domain, which precedes the SMP domain in sequence.

The membrane tethering properties of at least some SMP domain proteins are subjected to regulation. In the case of one E-Syt family member, E-Syt1, and of TMEM24, a key player in such regulation is cytosolic Ca^2+^, but interestingly in opposite ways. E-Syt1 is recruited to ER–plasma membrane contacts by cytosolic Ca^2+^ elevations via the Ca^2+^-dependent regulation of the bilayer binding properties of its central C2 domain (C2C) [54, 56]. In contrast, TMEM24 is present at contact sites when cytosolic Ca^2+^ is low, and redistributes throughout the ER when Ca^2+^ levels rise during signaling events [47]. This is due to the protein kinase C-dependent phosphorylation of its basic C-terminal region, which results in its acidification and thus in its shedding from the negatively charged plasma membrane bilayer. Subsequent dephosphorylation by calcineurin allows TMEM24 to return to contact sites, where it may participate in replenishing PI(4,5)P_2_ pools via its PI transport properties. PI(4,5)P_2_ regulates the activity of plasma membrane ion channels involved in Ca^2+^ dynamics and serves as a precursor for IP_3_, which stimulates Ca^2+^ release from the ER. Thus, TMEM24, in addition to being regulated by Ca^2+^, can also reciprocally participate in the regulation of cytosolic Ca^2+^ in cells where it is highly expressed, such as pancreatic ß cells [47].

In yeast, Nvj2 was reported to relocalize from the nuclear–vacuolar junction to ER–Golgi contacts in response to ER stress, thus pointing to regulation also for this SMP domain-containing tether, although the underlying mechanisms remain unclear [48]. Whether ERMES-mediated ER–mitochondria tethering undergoes regulation is not known. If so, it would have to involve disassembly of the complex, as two of its components are integral membrane proteins of the ER and of the mitochondria, respectively.

Much remains to be understood about SMP domain-containing proteins and their tethering and lipid transport functions. Their number is likely to expand, as the structural characterization of proteomes advances. Key open questions are the mechanisms through which these proteins extract and then deliver lipids from and to bilayers and the regulation of these reactions. As SMP-dependent transport between bilayers does not require energy and flows down the concentration gradient of the lipids, there must be mechanisms to control their lipid transport activities in order to preserve the heterogeneous lipid composition of participating bilayers. It also will be important to elucidate interplay of SMP domain proteins with other lipid transport modules and with membrane tethers that have functions unrelated to lipid transport, such as those that control Ca^2+^ dynamics. Given the Ca^2+^ regulation of some SMP domain proteins, cross-talk with membrane tethering proteins that regulate cytosolic Ca^2+^ is of special interest. The field of membrane contact sites is rapidly advancing and opening new vistas about inter-organelle communication in cellular function.

## Imaging approaches to study organelle interactions and dynamics

### Sarah Cohen, Alex M. Valm and Jennifer Lippincott-Schwartz

A hallmark of eukaryotic cells is their organization into membrane-bound compartments. This allows for the spatial and temporal separation of incompatible biochemical processes. Nevertheless, cellular organelles (including endoplasmic reticulum (ER), mitochondria, Golgi, lysosome, and peroxisome) must co-ordinate their activities to allow the cell to function properly as a biological system. An emerging theme is that communication between compartments often occurs at sites of close apposition between organelle membranes, called membrane contact sites (MCSs). These MCSs play important roles in metabolic channeling, allowing molecules such as lipids and calcium to be transferred directly from one organelle to another [60]. Identifying the proteins that mediate organelle contacts is an active area of research [61] and contacts between many different organelle types are being newly discovered. For example, VAMP-associated proteins (VAP)s on the ER have been shown to bind proteins with two phenylalanines in an acidic tract (FFAT) motifs, which are found on many other organelles [62]. Recently, a variety of new imaging techniques have been applied to elucidate the structure, function, and dynamics of MCSs.

MCSs have long been observed by electron microscopy (EM)—see the contribution by Tim Levine in this forum for a discussion of the history of organelle contacts. Modern electron microscopy techniques, including cryo-electron microscopy and tomography, have revealed a number of structural features of MCSs. It has been observed that the ER makes multiple discreet contacts with mitochondria and that an average mitochondrion has 2–5% of its surface involved in MCSs with ER [63]. Cryo-electron tomography revealed structural differences between different types of ER–plasma membrane contact sites. STIM1-Orai1-mediated contacts were spanned by filaments perpendicular to the ER and plasma membrane, while E-Syt-mediated contacts exhibited a smaller gap between membranes, and were not spanned by filaments [56] (see “Membrane tethering and lipid transport by SMP domain containing proteins” for further discussion of E-Syt-mediated contacts). Electron microscopy techniques allow visualization of MCSs with exquisite resolution; however, these techniques are not well suited to answer questions about organelle and MCS dynamics, because cells must be fixed prior to imaging.

The advent of genetically encoded fluorescent labels, such as green fluorescent protein (GFP) and its spectral variants, has revolutionized the study of cell biology and allows imaging of the dynamic processes in living cells, including MCSs. The use of fluorescent proteins targeted to the ER and other organelles facilitated further insights into the functions of MCSs—that of organelle remodeling. Work from the laboratory of Gia Voeltz demonstrated that fission of both mitochondria and endosomes occurs at sites of contact with the ER [64, 65]. How general a phenomenon this is remains to be demonstrated. Other fluorescence-based approaches have been used to obtain complementary information about MCSs. Förster resonance energy transfer (FRET) is a fluorescence imaging technique that identifies when two fluorophores are within 10 nm of each other (the FRET distance). Csordás et al. have used FRET pairs targeted to the ER and mitochondria to identify these MCSs in live cells, and demonstrated the remarkable result that all mitochondria make contacts with ER [66]. Bimolecular fluorescence involves the use of a split Venus fluorescent protein construct. One half of the Venus protein is conjugated to a membrane protein in one organelle, and the other half to a known and specific membrane protein in another organelle. Although the technique may bias the frequency and duration of MCSs by artificially stabilizing them, Schuldiner and colleagues have used this technique for its extraordinary power to discover MCSs between organelles not previously observed [61]. Bimolecular fluorescence complementation has also been used to investigate the interaction between specific proteins on different organelles, for example, between lipid droplets (LDs) and mitochondria or peroxisomes [67].

**Fig. 4.**
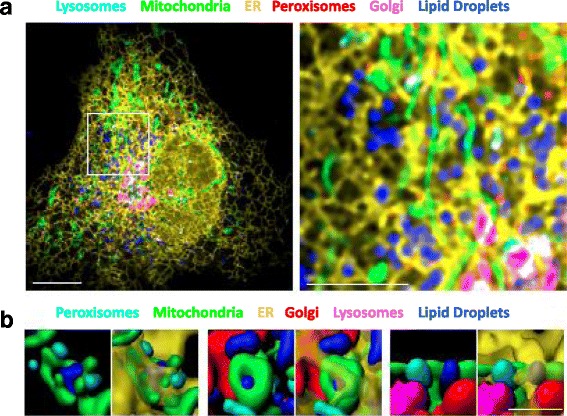
Spectral imaging reveals organelle interactions. **a** *Left*: micrograph of a COS-7 fibroblast cell expressing fluorescent fusion proteins LAMP1-CFP, mito-EGFP, ss-YFP-KDEL, mOrange2-SKL, and mApple-SiT, and labeled with BODIPY 665/676. Images were acquired on a confocal microscope with a spectral detector, and subjected to linear unmixing and spatial deconvolution. *Scale bar*, 10 μm. *Right*: enlargement of the region outlined in the *left panel. Scale bar*, 5 μm. **b** Examples of complex organelle contacts in segmented multispectral lattice light-sheet images. The ER (*transparent yellow*) is shown in the *right panels* only. *Scale bar*, 2 μm

Live cell fluorescence imaging is a powerful technique to answer systems-level questions about the frequency, duration, and overall dynamics of MCSs. However, the inability to distinguish fluorophores with highly overlapping emission spectra has limited its use to the labeling of a few different organelles in live cells. Our laboratory has recently developed a cell labeling, imaging, and computational analysis method to identify the systems-level dynamic organization of organelle interactions in cells [68]. We targeted fluorescent fusion proteins to ER, mitochondria, Golgi, lysosomes, and peroxisomes and used a vital dye to label LDs. We then used confocal and lattice lightsheet spectral imaging approaches to image these six organelles simultaneously, in live fibroblast cells (Fig. 4a). This approach provides a powerful tool to identify potential MCSs and to generate hypotheses regarding their dynamics. We demonstrated that all six labeled organelles made contacts with each and every other labeled organelle. These contacts often had elaborate morphologies, involving multiple different organelles in close proximity (Fig. 4b). We further demonstrated that these contacts were dependent upon an intact microtubule cytoskeleton, as treatment with nocodazole, which disrupts microtubule polymerization, reduced the number of organelle interactions, especially those involving LDs. Surprisingly, we observed that although individual organelles are highly dynamic, the sum of all organelle contacts in fibroblast cells is highly stable over time, forming a consistent pattern that we termed the ‘organelle interactome’. This pattern shifted in response to changes in the availability of nutrients (for example, starvation or excess fatty acids), but the ER always remained the central node in the interactome network, making the most contacts with other organelles.

We used this spectral imaging approach to track individual organelles, and map their contacts over time. We focused on LDs, which are thought to exchange lipids including phospholipids, fatty acids, and cholesterol with various other compartments in response to cell signaling events [69]. Tracking LDs, we found that most made near continuous contact with the ER, which is a major site of lipid synthesis. At any given time, approximately 85% of LDs were in close proximity to the ER, and some of these contacts were sustained over the entire five-minute imaging period. Contacts between LDs and other organelles were shorter, and occurred in various different combinations. Some LDs made contact with only one or two other organelles over the course of five minutes, while some LDs touched every other labeled organelle in rapid succession. We also visualized contacts between mitochondria and other organelles, and found that mitochondria make the most contact with ER, followed by Golgi and LDs. These dynamics provide clues about the coordination of function between certain groups of organelles, and may reflect the amount of metabolic flux between compartments at steady state, under various conditions (for further discussion on the coordination of lipid metabolism between peroxisomes and other organelles, see “Interorganellar dynamics drive peroxisomal biogenesis and function”). In the future, this method could be combined with probes that detect metabolites such as lipids or calcium, in order to measure their transfer between organelles at MCSs directly.

Multispectral and other imaging methods will be useful for investigating the effect of various perturbations on organelle contacts and dynamics. We envision using this approach to investigate organelle organization in response to various environmental and developmental cues, including changes in the availability of nutrients, exposure to drugs, and infection with pathogens. Organelle contacts are also likely to change over the course of the cell cycle, and may show unique signatures in different regions of polarized or migrating cells. Faster imaging will reveal even more information about the dynamics of different types of organelle interactions. Gentler imaging methods will allow organelles to be tracked over longer periods of time, which may reveal that the pattern of organelle contact changes over the course of an organelle’s lifetime. For example, LDs may interact with different compartments as they are born (emerging from the ER), grow, deliver lipids to other compartments, and turn over (via lipolysis or lipophagy). Spectral imaging will also be a useful tool for identifying proteins that mediate or regulate MCSs, and for visualizing protein complexes and cytoskeletal elements at the interface between organelles. MCSs are a fascinating frontier in cell biology, mediating a variety of important metabolic functions. We are confident that the continued development of better fluorescent probes, as well as ever gentler, faster and higher-resolution imaging methods, will allow for exciting new discoveries in this emerging field.

## The history of contact sites: a series of near-misses

### Tim P. Levine

Today intracellular communication at contact sites, places where two compartments make a ‘near miss’ with each other, is a hot topic. Here I will review some of the 60-year history of this field, to identify four of the landmark discoveries and also point out some intellectual near misses along the way.

1. *First Contact*. Even though there is no accepted definition for a contact site, the closer and more extended a proximity is, the more it suggests some function. This has meant that the contact sites with the narrowest gaps tend to have been studied most. And since these gaps can be as small as ~ 10 nm, the best tool to resolve them is the electron microscope. The first contacts were described in 1956 (Fig. 5a), when the French microscopists Bernhard and Rouiller found that both mitochondria and the plasma membrane make intimate contacts with the ergastoplasm, an early name for endoplasmic reticulum (ER) [70]. For no reason I can find now, this work was never cited by more prominent microscopists such as Palade and Porter (According to Google Scholar searches for Bernhard AND KR Porter [AUTHOR] or GE Palade [AUTHOR]). Working on muscle cells these authors reported similar contacts in 1957 [71].

**Fig. 5.**
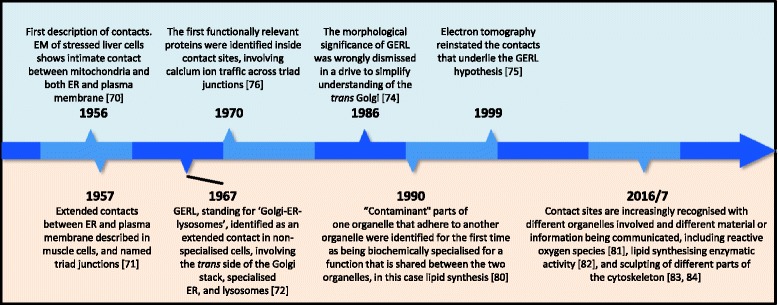
Timeline of early contact site discoveries. For references in the timeline please see [70–72, 74–76, 80–84]
2. *A refashioned GERL*. Ultrastructural analysis by electron microscopy progressed to the point where Alex Novikoff identified regions of specialised ER associated with Golgi membranes, particularly on the trans face of the Golgi near lysosomes. He named the whole region of the cell where these three organelles come together as GERL, standing for ‘Golgi-ER-lysosomes’. Novikoff proposed wrongly that lysosomes develop directly from the Golgi-associated ER [72, 73]. Because of this error in proposed function, the significance and accuracy of the morphological concept of contact between ER and trans-Golgi was easily dismissed [74]. Many years passed before the application of electron tomography by Richard McIntosh, Kathryn Howell and co-workers [73, 75] revealed an entire cisterna of so-called ‘trans ER’ sandwiched between cisternae of the trans side of the Golgi apparatus, showing that the original GERL hypothesis was morphologically correct. Subsequently, ER–Golgi contact sites have been ascribed roles in lipid traffic [14] (and see contribution by Guillaume Drin). Furthermore, interactions between organelles in this region of the cell are now known to be even more complex than Novikoff envisaged (see contribution by Sarah Cohen et al.).
3. *Giving the idea some muscle*. It is no accident that some of the easiest contacts to see are in muscle cells, as their highly enlarged ER (called the sarcoplasmic reticulum (SR)) forms extended contacts. The plasma membrane is also unusual, invaginating to form long transverse tubules. Each transverse tubule is sandwiched between a pair of parallel SR tubules, together forming a triad, as named by Porter and Palade [71]. Along the length of each triad, the SR forms multiple close contacts with the transverse tubule. In 1970 Clara Franzini-Armstrong identified proteins inside the contact zones (Fig. 5b) [76]. Over the following three decades these proteins were shown to consist of coupled calcium channels that convert the plasma membrane depolarisation spike to a rapid widespread release of SR calcium. The idea that contacts allow interplay of calcium signaling between closely apposing organelles has since been generalised to the transport of ER calcium into mitochondria [77], external calcium reaching the ER [78] and endo/lysosomal–ER calcium signaling (see contribution by Clare Futter and Emily Eden) [79].
4. *No to interfacial purity*. A major approach in the early days of membrane cell biology was cell fractionation. Membrane fractions, for example from density gradients, were specifically enriched in individual organelles and de-enriched in all others. However, some compartments (mainly ER) were very often present at low levels in all fractions. A blinkered view might describe this as contamination. But what if compartments adhere to each other for an important function? This was exactly the thinking of Jean Vance, who showed in 1990 that ER membranes adhering to mitochondria are enriched in the enzyme that makes the lipid phosphatidylserine that is destined to be transferred from ER to mitochondria (Fig. 5c) [80]. The idea that adherent ER is adapted for bidirectional lipid exchange caused a paradigm shift away from membrane contact sites being random contaminants. The suggestion that lipid transfer might occur at contacts has become increasingly accepted, and is the subject of many contributions in this forum by Guillaume Drin, Karin Reinisch and Pietro De Camilli, and Clare Futter and Emily Eden. Contact sites have a wider role in lipid traffic for organelle biogenesis, as described in contributions by Delphine Judith et al. (autophagosomes) and by Ayumu Sugiura and Heidi McBride (peroxisomes).
5. *Intracellular conversation and information transfer*. New ideas abound for the roles of inter-organellar contacts either in unidirectional traffic, for example of reactive oxygen species [81], or in reversible exchange, for example by various enzymes stretching out across contacts to find substrates [82–84]. On a different plane, another vital dimension for information exchange is us scientists sharing ideas and techniques. In the past this was a stumbling block, as researchers in traffic of lipid and calcium worked in ghettoised super-specialities, never citing each other’s work. Fortunately, conversation (contact) has increased in the past decade. The barriers are coming down; *vive la révolution*!

## Sterol transport

### David B. Iaea and Frederick R. Maxfield

Cholesterol is an essential component of mammalian cell membranes, and it plays a major role in determining the biophysical properties of membranes with effects on signal transduction, transport properties, and permeability. Cells have evolved homeostatic processes that maintain the cholesterol level of each organelle within a narrow range. However, significant differences in cholesterol distribution are maintained among cellular organelles. In the plasma membrane of mammalian cells, cholesterol is approximately one third of the lipids [85], but in the endoplasmic reticulum (ER) cholesterol accounts for only ~ 5 mol% of total lipids [86]. The endocytic recycling compartment (ERC) has high levels of cholesterol and contains about 30% of the total cholesterol in some cultured cells [87]. In general, cholesterol levels increase gradually in membranes from the ER to the plasma membrane on the secretory pathway [88]. We are interested in understanding how cholesterol levels are sensed in organelles and also how cholesterol moves among organelles.

Vesicular transport is one mechanism for shuttling sterols and other lipids between organelles. Eukaryotic cells maintain a high rate of vesicular trafficking among the secretory and endocytic organelles and the plasma membrane. For instance, in cultured fibroblasts it has been estimated that the entire plasma membrane is internalized with t_1/2_ of 15–30 min [89]. There are similarly rapid transport processes on the secretory and recycling pathways. Differences in cholesterol content must be continuously restored despite the rapid mixing of membranes associated with this vesicular transport.

**Fig. 6.**
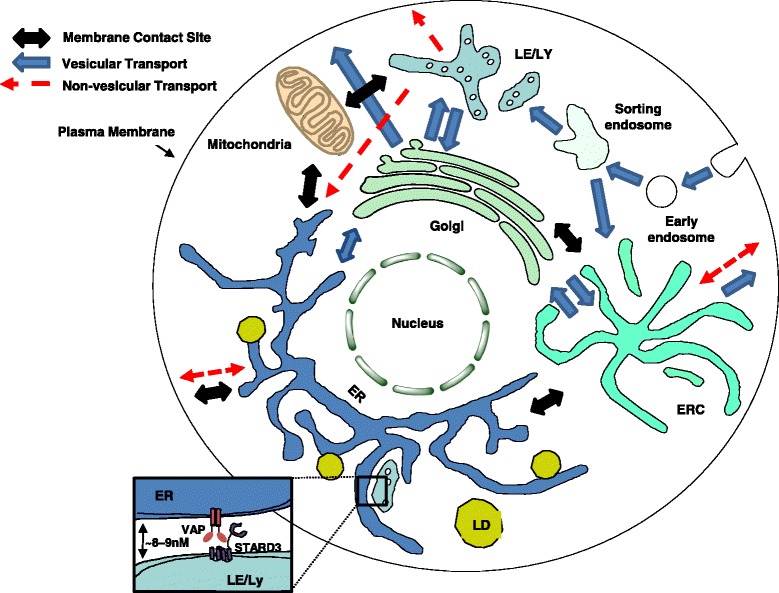
Sterol transport mechanisms. Vesicular transport processes are indicated with *solid blue arrows*. Non-vesicular transport processes are indicated with *red dashed arrows*. Membrane contact sites are shown as double-sided *black arrows*. The *inset* describes the membrane contact site formed between the ER and the LE/Ly [116]

For many years we have known that cholesterol and other sterols can move by non-vesicular transport mechanisms (Fig. 6). For instance, newly synthesized cholesterol is transported from the ER to the plasma membrane when vesicular trafficking is inhibited by either genetic or pharmacological intervention [90]. The ER is the main cholesterol sensing organelle in mammalian cells, and both lipoprotein uptake and cholesterol synthesis are regulated by the SCAP:SREBP-2 protein complex that resides in the ER [91]. However, only a small fraction of plasma membrane lipids move by vesicular transport from the plasma membrane to the ER, and specialized sorting processes are required [92, 93]. Thus, non-vesicular sterol transport would be required for the ER to respond rapidly and efficiently to levels of cholesterol in the plasma membrane and endosomes. Transport of cholesterol to the ER is also required for esterification of cholesterol by the ER resident enzyme acyl-CoA: cholesterol acyl-transferase (ACAT) [94]. The rate-limiting step in esterification of cholesterol by ACAT is delivery of cholesterol to the ER. ACAT provides a rapid high capacity mechanism for dealing with cholesterol excess, while the SREBP-2 mechanism maintains overall cholesterol homeostasis. These modes of regulation require cholesterol levels sensed in the ER to reflect the cholesterol distribution in other organelles, such as the plasma membrane and endosomes. To meet this requirement there must be a mechanism for rapid redistribution of cholesterol from these organelles to the ER.

Cholesterol can spontaneously desorb from membranes, but the rate is much too slow for the required transport among organelles [95]. There are several families of lipid transport proteins, and several members of these families can facilitate transfer of sterols between membranes. Two major gene families of lipid transfer proteins are the steroidogenic acute regulatory protein (StAR)-related lipid-transfer (START) domain family [96, 97] and the oxysterol binding protein (OSBP) and OSBP-related protein (ORP) family [98–100]. Among the START family of proteins, STARD4 has been implicated in maintaining sterol homeostasis and is transcriptionally regulated by SREBP-2 [101]. We are just starting to obtain information on the specific roles of these proteins in cholesterol transport and homeostasis.

Dehydroergosterol (DHE), a fluorescent sterol that mimics many properties of cholesterol [102], has been used to measure the distribution and transport kinetics of sterols in cells [87, 103]. Cholesterol can flip rapidly between the leaflets of the bilayer [104, 105], and it is enriched in the cytoplasmic leaflet of the plasma membrane [106, 107]. The abundance of cholesterol in the cytoplasmic leaflet may be one factor contributing to rapid non-vesicular transport among organelles. Using DHE it was found that sterol moves into or out of the ERC with a t_1/2_ of about 15 minutes in U2OS human osteosarcoma cells [108]. These rates of transport were only slightly reduced by ATP depletion, indicating that approximately 30% of sterol transport between these organelles is mediated by vesicular transport [108].

Non-vesicular transport between organelles can be accelerated by increasing the abundance of sterol carriers in the cytoplasm [103, 108]. Aside from an increase in cholesterol esterification by ACAT, there is little difference in the sterol distribution among organelles even when the rate of transport is increased five-fold [103, 108]. This suggests that the unequal distribution of cholesterol among cellular membrane compartments is not kinetically limited. The unequal sterol distribution can be attributed to the differences in the cholesterol–lipid interactions resulting from the differences in organelle lipid compositions [34, 109, 110]. The plasma membrane is relatively enriched in lipids that can stabilize cholesterol in the bilayer, while the ER is enriched in unsaturated lipids that provide weak stabilization of cholesterol. Because of these differences in cholesterol stabilization, these membranes can have unequal cholesterol concentrations even if sterol transporters bring them close to chemical equilibrium with one another [34, 109–111].

Recent studies show that STARD4, a soluble cytoplasmic transport protein, plays an important role in trafficking of cholesterol between several organelles, including the plasma membrane, ERC, and ER [103, 112]. Studies using U2OS cells demonstrated that STARD4 overexpression increased cholesterol ester levels and accelerated sterol transport between the ERC and plasma membrane [103]. STARD4 silencing attenuated cholesterol-mediated regulation of SREBP-2 activation [103]. Additionally it was recently reported that STARD4 in U2OS cells accounts for ~ 25% of total sterol transport and ~ 33% of non-vesicular sterol transport between the plasma membrane and ERC [108]. The quantitative role of other proteins in this transport is not known at present.

It has been estimated that approximately 10^6^ sterol molecules per second enter or leave the ERC in mammalian cells [113], but corrections for more recent measurements of transport rate [108] would indicate that the rate is closer to 2 × 10^5^ per second. This requires high levels of sterol transport proteins. In liposome transport studies it has been found that STARD4 can transport about 0.1 molecules of DHE per second [103]. STARD4 has been reported to interact with several organelles and is highly expressed in many cell types [103, 108, 114]. There are about 250,000 STARD4 molecules in a U2OS cell [108]. These rough estimates would be consistent with observations that STARD4 transports ~ 25% of the sterol between the plasma membrane and the ERC.

Several sterol transport proteins have been shown to operate at membrane contact sites, which provide a means to compartmentalize sterol transport between specific organelles. For instance, Osh4 [7] and oxysterol binding protein (OSBP) [9] have been reported to be modulated by a specific lipid, phosphatidylinositol-4-phosphate (PI4P). Both of these lipid transfer proteins exchange sterol in the ER for PI4P in the Golgi, generating a gradient to enrich sterol at the expense of PI4P [2, 115]. In this way, the enrichment of PI4P on the Golgi drives the vectorial transport of sterol from the ER to the Golgi. This mechanism of vectorial transport of lipid by exchanging with PI4P is not unique for sterol and has been recently described for phosphatidylserine transfer mediated by ORP5/8 [6] and Osh6/7 [10]. In addition to sterol gradient formation, membrane contact sites may also participate in maintaining cholesterol homeostasis. This is most notable in work involving late endosome/lysosome (LE/Ly) contact sites that may facilitate transport out of the LE/Ly, by STARD3, for direct deposition into the ER (Fig. 6) [116]. This mechanism would closely tie the degradation of lipoproteins, occurring in the LE/Ly, to homeostatic sterol sensing machinery in the ER. The role of several other membrane contact sites in sterol transport between organelles has been reviewed recently [117].

The molecular mechanisms by which transport proteins facilitate movement of cholesterol from one membrane to another are not well understood. As a first step, the very hydrophobic cholesterol must be removed from the bilayer, so a high free energy barrier must be overcome for desorption to occur. Sterol transfer proteins can interact directly with the membrane, reducing the energetic barrier and facilitating sterol removal from the membrane [118–121]. For several transport proteins, recruitment is mediated in part by electrostatic interactions and further mediated by insertion of a hydrophobic segment into the membrane bilayer [121]. These interactions likely result in local membrane perturbations that decrease sterol–phospholipid interactions and reduce the barrier for absorption of sterol into the core of the sterol transport protein. Following binding of sterol, the sterol transport protein releases from the donor membrane to move, in complex with the sterol, to deliver it to an acceptor membrane. For soluble proteins like STARD4 this presumably involves diffusion through the cytoplasm and contact with the acceptor membrane. For transporters in membrane contact sites, the proximity of the membranes may facilitate rapid exchange of sterol between the organelles by reducing the distance that the sterol–protein complex must travel [122]. However, it has been suggested that desorption of the cholesterol from the bilayer is the rate determining step in sterol transport, and diffusion of a small transport protein over the distances in a typical cell would not contribute greatly to the overall transport rates [118]. This analysis suggests that membrane contact sites can improve the targeting of sterol transport, but they would not greatly increase the rate of transport.

Cholesterol is a highly dynamic lipid heterogeneously distributed among organelles. It has rapid lateral and transverse mobility with dramatic effects on the organization of its surrounding lipids. There is increasing evidence that the majority of sterol trafficking and distribution is maintained by non-vesicular transport mechanisms. The presence of numerous sterol transport proteins provides multiple mechanisms that cells can use to rapidly redistribute sterol among organelles. We are just starting to understand the role of specific proteins in these processes, and we have little understanding of the molecular mechanisms by which transport proteins extract cholesterol from one membrane and deliver it to another. While there is still much work to be done, rapid progress is now being made in this field. An important question for the next few years is to elucidate the trafficking kinetics of sterols into and out of organelles and how these transport steps are integrated into the overall cellular homeostasis.

## Regulation of MVB biogenesis by ER–endosome membrane contact sites

### Clare E. Futter and Emily R. Eden

**Fig. 7.**
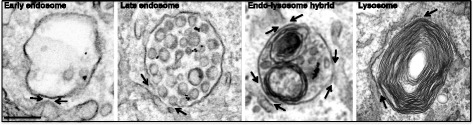
Membrane expansion during maturation of the endocytic pathway. Electron microscopy of cells stimulated with EGF and anti-EGFR-Au for 20–40 min. Examples show increased MCSs accompanying the gain in membrane content of endocytic organelles during maturation. *Black arrows*, ER contact sites. *Scale bar*, 200 nm

The endocytic pathway, by which cells internalize, sort and degrade a wide range of proteins and lipids, regulates essential cellular processes such as nutrient uptake, membrane homeostasis and signal transduction, as well as inter-cellular communication. Sorting events at the early endosome define the destination of endocytosed cargo, which can be recycling to the plasma membrane, retrograde transport to the Golgi, or delivery to the lysosome for degradation. As discussed in Tim Levine’s contribution to this forum, the possibility of physical associations between the endoplasmic reticulum (ER) and the endocytic pathway was first evoked some 50 years ago by researchers describing GERL (Golgi-associated smooth endoplasmic reticulum implicated in formation of lysosomes) [72], but only in the past decade have we begun to appreciate the fact that the ER makes multiple membrane contacts with the endocytic pathway that have central roles in the regulation and co-ordination of endocytic trafficking. MCSs (defined as sites where inter-organellar distance is < 30 nm) increase during endosome maturation (Fig. 7), with 90–99% of late endosomes and lysosomes estimated to form an ER contact site [123, 124] and also regulate endosomal positioning [25, 125]. Indeed MCSs are emerging as master coordinators of positioning, maturation and function of endocytic organelles, but here we will focus on the role of MCSs in multivesicular body (MVB) biogenesis and downstream regulation of EGF receptor (EGFR) tyrosine kinase signaling.

MVBs are defined by the presence of intraluminal vesicles (ILVs) within the endosome, or MVB, that form by inward budding of the limiting membrane as the endosome matures. Ubiquitinated cargo, such as EGF-stimulated EGFR, is targeted onto ILVs for later delivery to the lysosome by a series of protein complexes that comprise the endosomal sorting complex required for transport (ESCRT) machinery [126]. The first ESCRT complex, ESCRT-0, engages ubiquitinated EGFR and concentrates it in clathrin coated domains of the limiting membrane of the endosome. Following recruitment of subsequent ESCRT complexes, the EGFR is sequestered onto ILVs, removing the active receptor tyrosine kinase domain from cytosolic substrates and thereby attenuating receptor signalling. Multiple populations of MVBs have been described, which are defined by different membrane proteins and/or cargo. MCSs between the ER and different MVB populations are biochemically distinct in terms of their tethering complex composition. The subpopulation of MVBs that contain EGFR form ER contact sites that are specifically tethered by annexinA1 and its calcium-dependent ligand S100A11 [127]. AnnexinA1-regulated contact sites allow the ER-localised phosphatase, PTP1B, to mediate effects at the endosome. PTP1B dephosphorylates endocytosed EGFR and the ESCRT0 proteins, Hrs [128] and STAM [129], implicating MCSs in the regulation of ESCRT-dependent sorting. Indeed EGF-stimulated ILV formation is dependent on both PTP1B activity [128] and the MCS regulator annexinA1 [127, 130], suggesting that PTP1B-mediated dephosphorylation of Hrs at MCSs could modulate its function in ILV formation and therefore MVB biogenesis. Hrs, which is phosphorylated following EGF stimulation, has multiple roles at the endosome, including clathrin-dependent concentration of ubiquitinated cargo, recruitment of ESCRT-1, recycling of some endocytosed receptors, and cholesterol transport from endosomes to the ER. Which of these roles might require Hrs dephosphorylation by PTP1B is not known and neither is how clathrin coat and MCS assembly/disassembly are co-ordinated. The close apposition of membranes at an MCS precludes the presence of a clathrin coat. At least local disassembly of both clathrin coat and MCS must occur before ILV formation since neither clathrin nor ER are incorporated onto ILVs. Coated domains are sometimes found adjacent to the MCS [131] and it is tempting to speculate that the temporal coordination of clathrin coat, MCS and ILV formation could be regulated by modulation of the phosphorylation state of Hrs at MCSs.

The increase in ILVs as the endosome matures occurs without any apparent loss in endosome volume, suggesting a mechanism for endosomal membrane expansion (Fig. 7). This membrane is rich in cholesterol [132] which, together with the oxysterol binding protein ORP1L, is required for MVB biogenesis [133] and ILV formation [127]. Endosomal cholesterol can be derived from endocytosed low density lipoprotein (LDL), which is hydrolysed in the acidic environment of the endosome to release free cholesterol. However, when cells are cultured in the absence of LDL, they instead derive the cholesterol necessary for ILV formation from the ER. ER-localised VAPs are implicated in tethering ER contact sites with many organelles [127, 134], but are not required for the formation of the ER contacts with EGFR-MVBs that are regulated by annexinA1. However, although not required for their formation, VAPs do appear to function at ER MCSs with EGFR-MVBs since an interaction between VAPs and endosomal ORP1L at MCSs mediates the transport of ER-derived cholesterol to the MVB [127].

This interaction depends on ORP1L’s FFAT motif, the accessibility of which is increased under conditions of low endosomal cholesterol due to a conformational change in ORP1L on sterol binding [125]. Similarly the sterol-binding endosomal protein STARD3 was recently shown to promote sterol repartitioning to the endosome at MCSs [135]. Like ORP1L, in addition to sterol binding, STARD3 also functions in scaffolding the MCS through interaction with VAP [136]. STARD3 and ORP1L localise to different endosomal populations [137] and may function independently, with STARD3 mediating cholesterol transport in the absence of EGF stimulation, most likely to EGFR-negative MVBs, while ORP1L promotes cholesterol transport to EGFR-positive MVBs under conditions of low LDL. Thus, consistent with multiple biochemically distinct populations of ER–endosome MCSs, STARD3 and ORP1L appear to operate in parallel mechanisms of sterol transport at ER–endosome MCSs.

Lipid repartitioning at MCSs might also facilitate the fission of tubular buds for recycling and retrograde transport. ER–endosome MCSs correlate spatially and temporally with the sites of membrane constriction and fission of recycling tubules, whilst also defining sites of fission for cargo destined for the Golgi. These sites are marked by FAM21, a retromer-binding subunit of the WASH complex [65]. The mechanism by which MCSs define endosomal fission events is not entirely clear but a recent study implicates the MCS-mediated regulation of phosphoinositide distribution in this process [134]. When MCSs were disrupted by loss of VAPs, a marked increase in the endosomal pool of PI4P resulted, due to reduced dephosphorylation by the ER-anchored phosphatase Sac1. MCSs thus provide sites for Sac1-mediated PI4P dephosphorylation, either following PI4P transport across the contact to the ER or *in trans* at the MCS. Since VAPs are also required for transport of ER-derived cholesterol to the endosome, this sterol transport might be mechanistically similar to the OSBP-mediated sterol/PI4P exchange that occurs between the ER and the Golgi [9] that is discussed in more detail in Guillaume Drin’s contribution to this forum. Thus, MCSs serve a dual role in ILV formation, by providing both sites for PTP1B-mediated effects on the ESCRT machinery and platforms for lipid transport to support membrane expansion. Interestingly, another phospholipid, phosphatidylserine (PS), was recently shown to be transported at MCSs, with newly synthesized PS transported from the ER to the endosome [138]. In yeast PS is converted at the endosome to phosphatidylethanolamine (PE), which plays an important role in endosomal fusion events [139].

Endosome–lysosome fusion to form a hybrid endo-lysosomal organelle allows endosomal cargo to be degraded and is followed by reformation of the lysosome [140]. Both endosome–lysosome fusion and lysosome reformation are regulated by Ca^2+^ [141]. ER–lysosome MCSs have been implicated in the generation of localised Ca^2+^ signals that likely promote these events. Mobilization of lysosomal Ca^2+^ stores evoked release of Ca^2+^ from the ER resulting in amplification of the lysosome Ca^2+^ signal [124]. This coupling of Ca^2+^ release is most likely mediated by MCSs between the ER and lysosomes [124] and is bidirectional, with ER Ca^2+^ store release inducing a gain in lysosome pH that is reflective of Ca^2+^ loss [142]. Lysosomal Ca^2+^ release is mediated by two types of Ca^2+^ channel, the NAADP-sensitive two pore channels (TPCs) and the mucolipins (also called TRPMLs). Both have been implicated in coupling Ca^2+^ release from acidic stores and the ER TPC1 [143] was recently shown to localise to ER contacts with MVBs and to regulate their formation and consequent PTP1B-mediated down-regulation of EGFR signalling [79]. This raises the intriguing possibility of reciprocal regulation of Ca^2+^ signalling and MCSs between the ER and endocytic pathway. Although the precise role of TPC1 activity on transport through the endocytic pathway remains to be characterised, inhibition of TPC1 activity resulted in changes in morphology and perinuclear clustering of late endosomes and lysosomes [79]. Thus, Ca^2+^ signalling at ER–endocytic pathway MCSs may be a key component of the regulation of endosomal positioning, traffic and function by MCSs.

How traffic in the endocytic pathway is co-ordinated has been a subject of much speculation and debate for more than three decades. The identification of MCSs shed new and unanticipated light on this topic by implicating another organelle, namely the ER, in this co-ordination. That there are multiple populations of biochemically distinct MCSs between the ER and endocytic pathway that are differentially regulated lends considerable support to the notion that MCSs have a central role in co-ordinating the formation and function of the endocytic pathway.

## Autophagosome biogenesis: what’s the source of the phagophore membrane?

### Delphine Judith, Alexander R. van Vliet, Patrizia Agostinis, Sharon A. Tooze

From fasting humans to starving cells, from muscle atrophy to mitophagy, the ability to digest one’s self is a recurring and crucial aspect of eukaryotic life. In a nutshell, macroautophagy (here referred to as autophagy) constitutes the engulfment by a double membrane of intracellular compartments or organelles that will be digested and recycled by the cell.

The first critical event in this pathway is the nucleation of a membrane called the phagophore or isolation membrane. It can expand and grow through the acquisition of lipids, trapping a part of the cytoplasm containing various components marked for destruction. This entrapment can be selective or non-selective. The phagophore is an unusual membrane with unique morphological properties. Using conventional EM preparation techniques it appears as a double-membrane with a cup-shaped form that stains heavily with osmium tetroxide, while the lumen appears semi-transparent. Moreover, using freeze-fracture approaches the double membrane was shown to be protein-poor. These peculiarities suggest that its lipid and protein composition is exceptional and distinctive from other cellular membranes. Ever since autophagy was discovered in the 1950s, much work has gone into uncovering the intricate mechanism and interplay of autophagy-related (ATG) proteins implicated in autophagosome formation. However, important and seemingly simple questions about this pathway have so far eluded researchers around the world. What is the origin of the phagophore membrane? Where do the phagophore membrane and then the autophagosome receive most of their lipids? Emerging answers to these fundamental questions have revealed a vast web of organelles, all contributing major constituents to the autophagosome.

**Fig. 8.**
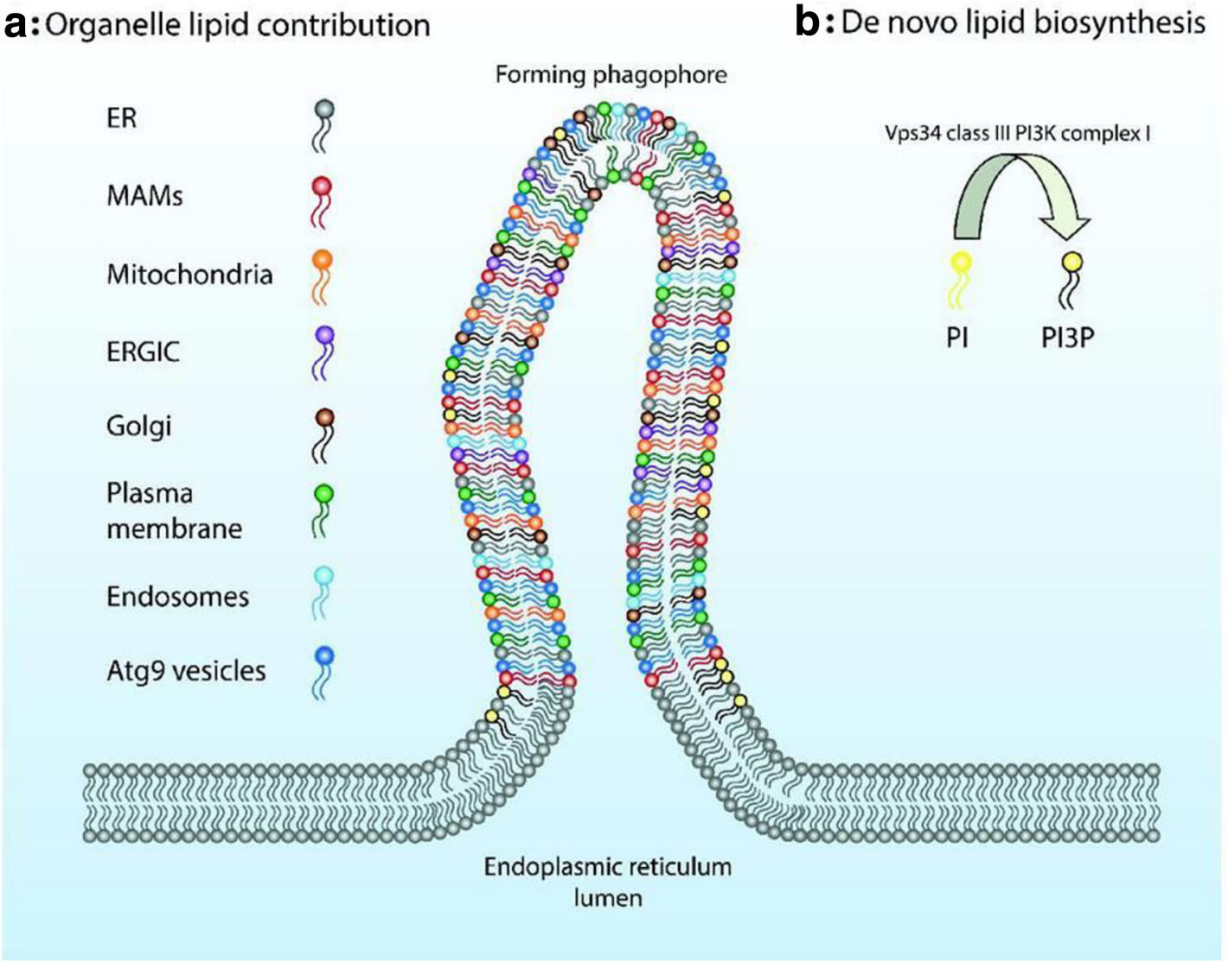
Overview of the lipid origin for the formation of the phagophore membrane. **a** The lipids of the forming phagophore membrane originate from multiple sources such as the ER, mitochondria, Golgi, plasma membrane and other compartments but also from **b** de novo synthesis of PI(3)P by the Vps34 PI3K kinase complex I

It therefore comes as no surprise that there is no broad consensus about the various sources of the phagophore membrane. Studies have revealed that it arises from a specific membrane structure that originates from the endoplasmic reticulum (ER) membrane, known as the omegasome. The omegasome is the earliest visible autophagy structure and is proposed to function as a platform for autophagosome formation [144]. However, this theory became muddled by the observation that mitochondria are able to contribute membranes to the growing autophagosome [145]. Intriguingly, these two theories might have been neatly merged by the observation that autophagosomes form at ER–mitochondria contact sites, also known as mitochondria-associated membranes (MAM) [146]. The MAM is a crucial site of calcium trafficking between the ER and mitochondria, and a hotspot of lipid synthesis. Despite the observation that these two organelles, and their crossroads, are suggested to be the main platform for autophagosome biogenesis, the story is still complex. Studies have shown that the Golgi apparatus, plasma membrane and ER-Golgi intermediate compartment (ERGIC), but also the endosomal pathway, are able to contribute to membrane expansion and thus lipid delivery during the growth of the phagophore [147]. Finally, there is the potential role of the elusive ATG9 vesicles containing the only multi membrane spanning ATG protein, ATG9. While their exact function is not entirely clear, we know that they are crucial for autophagy. ATG9 vesicles make transient contact with the growing autophagosome, possibly delivering proteins and lipids to its membranes [148] (Fig. 8a).

One of the reasons that the MAM was found to be a site of autophagosome biogenesis was the recruitment of the Vps34 class III PI3K kinase complex I, containing ATG14, to this site upon starvation. The local enrichment of phosphatidylinositol 3-phosphate (PI3P) is a crucial step on the path to autophagy, since the omegasome is formed at specific PI3P-enriched ER subdomains (Fig. 8b). This subdomain acts to recruit effector proteins and stabilises the essential ATG complexes at the autophagosome formation site. PI3P is a cone-shaped membrane lipid that may lead to cytosol-facing budding of the membrane, which facilitates binding of proteins that sense membrane curvature. A specific ATG protein, WIPI2b, binds to PI3P to aid the progression of autophagy [149]. Whether the PI3P itself, produced in the ER environment, has a unique function is still up for debate. To add to the complexity, besides the production of PI3P, essential at the primary stage of autophagosome formation, the proper turnover of the omegasome-specific lipids must occur at a later stage of the process [150].

While many aspects of autophagosome biogenesis, from its origin to the mode of initiation, are quite well understood, a multitude of challenges remain to be resolved. The consensus is that PI3P is the most important and crucial lipid guiding the initial steps of autophagy. But what do we know about the other phosphorylated phosphatidylinositols? Are PI4P, PI5P, PI(3,5)P_2_ and PI(4,5)P_2_ in the picture? Recent research has been shedding light on their role in autophagy but much remains to be solved [151]. Moreover, the presence of specific lipid modifiers like kinases and phosphatases may permit lipid conversion at the phagophore itself. Autophagosome biogenesis is thus a complex picture with many different players, all of whom contribute small pieces of the puzzle. Understanding them all is crucial to understand the entire process of autophagy and potentially harness the pathway in a therapeutic setting.

## Interorganellar dynamics drive peroxisomal biogenesis and function

### Ayumu Sugiura and Heidi M. McBride

**Fig. 9.**
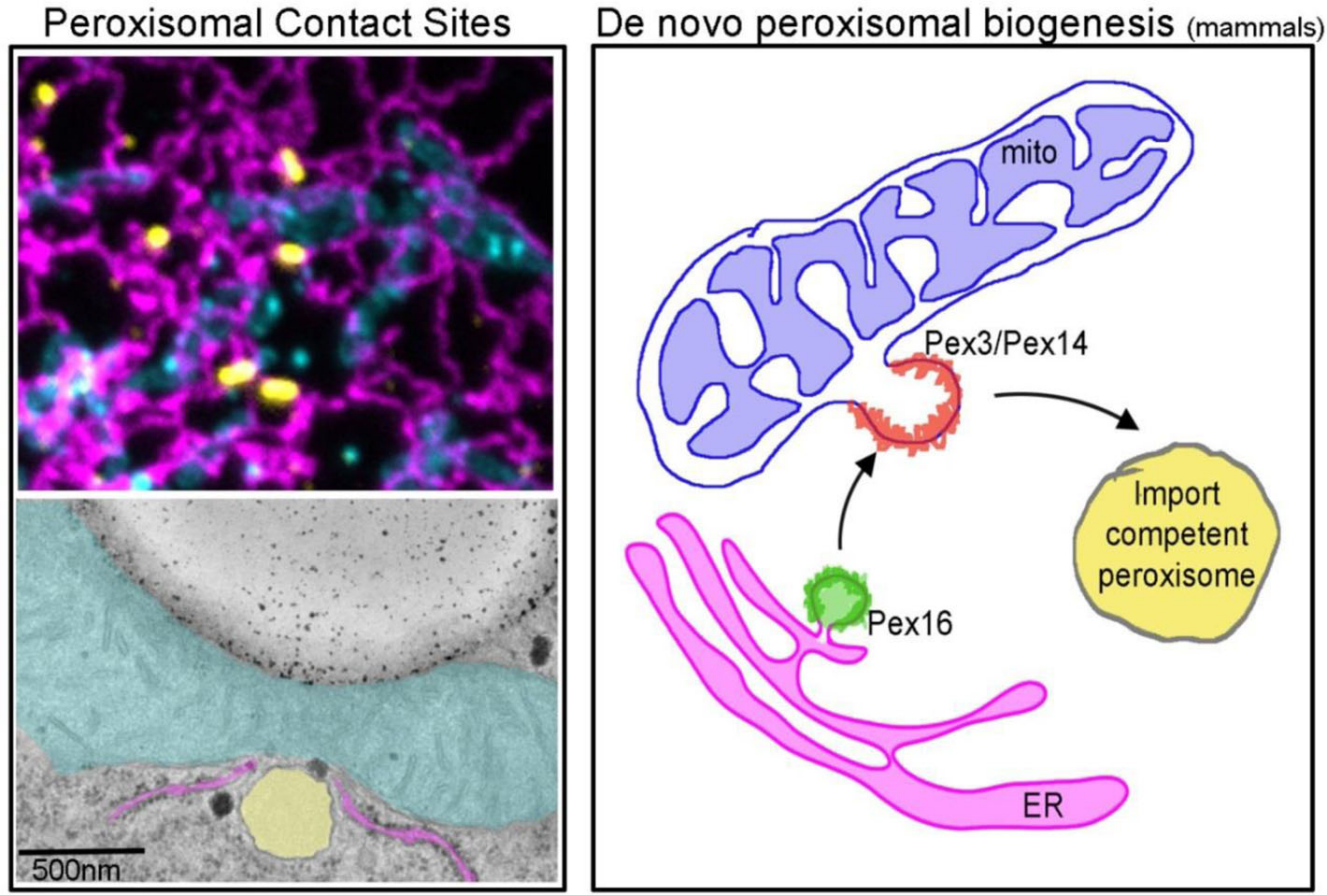
Peroxisomal biogenesis through interorganellar contact sites and transport pathways. Peroxisomal contact sites (*left box*, *top panel*) shows a confocal image of peroxisomes (*yellow*, anti-PMP70) in contact with ER (*magenta*, anti-KDEL) and mitochondria (*blue*, anti-TOM20) within a COS7 cell. These mammalian contacts were shown to require ACBD5 and VapB, as described in the text. *Bottom panel* shows an electron micrograph of a mouse liver cell illustrating direct contacts between a peroxisome (*yellow*), the ER (*magenta*) and a mitochondria (*blue*). A lipid droplet is also seen in direct contact with the mitochondria (on *top*). Many metabolites are modified by enzymes within multiple compartments, particularly bile acids within the liver that use the ‘catalytic triad’ of organelles seen here between the ER/mitochondria and peroxisomes. The emerging model of de novo peroxisomal biogenesis in mammalian cells is shown in the *right panel*. Pex16 is targeted to the ER, whereas Pex3 and Pex14 target the mitochondria in Zellweger patient fibroblasts lacking peroxisomes. Each is sorted into vesicular profiles that merge to form an import-competent peroxisome that continues to grow and divide. The molecular mechanisms and regulation of peroxisomal contact site formation and biogenesis are only just beginning to be understood

Peroxisomes are single membrane bound organelles that house a host of biochemical reactions, from the ubiquitous reduction of peroxide to the production of bile acids in the liver. The biochemical reactions occurring within peroxisomes are often dependent on contacts with other organelles [152]. Most peroxisomal enzymes perform their reactions as part of a more complex enzymatic cascade involving additional steps that occur within mitochondria and/or the endoplasmic reticulum (ER). Therefore, the completion of these biochemical pathways requires the active transport of metabolic intermediates between organelles, a process increasingly viewed to occur through the dynamic assembly of contact sites [152]. Common examples include the peroxisomal oxidation of very long or branched chain fatty acids, whose products are then transported to mitochondria for complete oxidation. Similarly, the production of plasmalogen is initiated in peroxisomes and then completed in the ER [153]. Peroxisome biogenesis can occur through both the growth and division of pre-existing organelles, or through de novo formation of nascent peroxisomes [154] (Fig. 9). Like mitochondria, peroxisomal numbers are governed primarily through growth and division cycles, where proteins are targeted through conserved import machineries. Peroxisomal biogenesis also requires other organelles to obtain lipids to remodel membrane and membrane itself as a source for de novo synthesis. Here we will outline the association between peroxisomes and other organelles by both direct tethering and vesicle transport.

To begin, the expansion of peroxisomal membrane requires the acquisition of lipids, first shown to occur through direct contact sites with the endoplasmic reticulum [155]. While the mechanisms underlying these contacts are still emerging, work in *Saccharomyces cerevisiae* and *Pichia pastoris* has shown that ER-localized Pex30 is restricted to peroxisomal contact sites, where it functions along with a number of partner proteins to modulate peroxisomal biogenesis and morphogenesis [156–158]. Functional inter-organellar contact sites that facilitate lipid or ion flux occur when the two organelles are brought within 15–30 nanometers through the action of membrane tethering machinery; however, in the case of Pex30, evidence for a role as a direct tether is lacking.

Most recently, two independent studies in mammalian cells identified the ER-localized VAP-A/B as a tether that binds the acyl-coA binding domain protein 5 (ACBD5) on peroxisomes, bringing the organelles into close proximity [159, 160]. VAP-B is a membrane anchored adaptor protein that binds a host of cytosolic proteins containing a conserved FFAT domain (two phenylalanines in an acidic tract), many of which act as lipid exchangers between the ER and a number of different organelles [62]. Indeed, silencing these tethering factors leads to a reduction of plasmalogen and cholesterol [159, 160]. These studies experimentally confirmed the importance of direct ER/peroxisomal contact sites for lipid exchange to promote peroxisomal biogenesis and metabolic lipid flux, marking a watershed moment in our understanding of the molecular machinery that governs peroxisomal contact site formation in mammalian cells. Importantly, mutations in ACBD5 and VAP-B are linked to retinal dystrophy and white matter disease [161, 162] and amyotrophic lateral sclerosis [163], highlighting the potential importance of these contacts in disease.

An unexpected role for contact sites between peroxisomes and lysosomes in intracellular cholesterol transport was recently described [164]. LDL-derived cholesterol was transported from the lysosome to the peroxisome in a manner that depended upon lysosomal synaptotagmin VII (Syt7) binding to PI(4,5)P_2_ on the peroxisomal membrane. These dynamic contact sites between lysosomes and peroxisomes were shown to depend upon the lysosomal cholesterol transport proteins Neiman-Pick type C proteins (NPC1 and NPC2). As with ACBD5 and VAP-B, mutations in these two proteins are linked to Neiman-Pick diseases [165]. Loss of NPC1 or NPC2, as well as a number of peroxisomal proteins, led to cholesterol accumulation in the lysosome [164].

Direct contacts have also been established between peroxisomes and mitochondria. Genome-wide screening approaches in yeast identified Pex11 and Mdm34 as potential tethers between peroxisomes and mitochondria, although the functional impact of these contacts was not clear [166]. Mdm34 is a component of the ER–mitochondrial encounter structure (ERMES), indicating common machineries modulating ER–mitochondria and mitochondrial–peroxisomal contacts [50]. In mammals, although peroxisomes share various molecules and pathways with mitochondria, a factor tethering them remains to be identified. Additional studies have documented the contribution of lipid droplets as a source of lipids for peroxisomal biogenesis and in the exchange of fatty acids and other lipids for metabolism; however, the machineries that regulate this remain elusive [167, 168]. With the emergence of new machineries and mechanisms governing interorganellar contacts, the peroxisome represents an excellent model organelle to study complex, multi-functional contacts with distinct organelles. The dynamics and regulation of these contacts are critical for both biogenesis and cellular metabolism.

Lastly, peroxisomes are also integrated in vesicular transport pathways at multiple levels. A contribution of ER and mitochondrial-derived vesicles has been proposed to deliver lipids and proteins to either generate peroxisomes de novo, or contribute to the pre-existing peroxisomal pool [169–173]. Yeast model organisms show significant plasticity in the generation of newly born peroxisomes, which can be promoted experimentally through growth on lipid sources like oleate [174]. In yeast lacking peroxisomes, a number of core peroxisomal membrane proteins target the ER and are enriched within pre-peroxisomal vesicles that fuse to form import-competent peroxisomes that subsequently grow and divide [175, 176]. Even in the growth and division phase, yeast peroxisomes have been shown to receive vesicles from the ER carrying specific proteins and, likely, lipids [173]. Recent studies in mammalian cells have expanded the model for de novo peroxisomal biogenesis [177]. This study used patient-derived fibroblasts lacking the core import receptors Pex3 or Pex16, rendering the two cell lines completely devoid of peroxisomal membranes. Upon rescue with the missing peroxin, it was shown that, in contrast with yeast model systems, Pex3 was imported into mitochondria where it exited in pre-peroxisomal vesicles that also contained endogenous Pex14 [177]. While Pex3 is essential for the import of peroxisomal membrane proteins, Pex14 is an established import receptor for luminal peroxisomal protein import. Therefore, multiple components of the peroxisomal import machinery are first targeted to mitochondria in the absence of peroxisomes. In contrast, the essential import receptor Pex16 targeted the ER before exiting in vesicles that fused with the mitochondrial-derived pre-peroxisomes, thereby generating a fully import competent, newly born peroxisome. The hybrid nature of de novo peroxisomal biogenesis hints at additional levels of co-ordinated signalling to initiate peroxisomal expansion. However, the major question remained as to the extent and regulation of de novo peroxisomal biogenesis in vivo. It was shown that Pex3 import into mitochondria was initiated in wild-type fibroblasts following the activation of pexophagy or the autophagic degradation of peroxisomes [177], highlighting the competence of these wild-type cells to initiate de novo biogenesis following a physiological trigger.

As in yeast [173], evidence exists in mammalian cells for the steady state delivery of vesicles from both the ER and mitochondria to peroxisomes, providing selected proteins and lipids [169–172, 178]. Early studies identified a vesicular transport route to deliver at least one mitochondrial membrane protein, a SUMO/ubiquitin E3 ligase MAPL/MUL1, to peroxisomes [172]. Cargo selection into vesicles was mediated by the retromer complex Vps35/Vps26/Vps29 and shown to be constitutive, suggesting a mitochondrial contribution to the peroxisomal pool in steady state [171, 179]. However, loss of Vps35 did not significantly affect peroxisomal numbers and did not inhibit de novo biogenesis [171, 177]. This indicates that steady-state delivery of MAPL/MUL1 is not essential for peroxisomal growth. These data provide an interesting mechanistic distinction between the mitochondrial-derived vesicles (MDVs) that generate pre-peroxisomes for de novo biogenesis (~200 nm, single membrane vesicles) and the MDVs that constitutively carry cargoes to pre-existing peroxisomes (~70–120 nm double-membrane vesicles) [172, 177]. Together these data hint that the insertion of at least some peroxisomal membrane proteins into the bilayer utilize the machineries within the ER and mitochondria, rather than the peroxisomal import complexes. Overall, although peroxisomes are equipped with an autonomous protein import apparatus, they remain dependent on dynamic interactions with other organelles, both through contact site formation and vesicle transport, for growth.

The last few years have seen the identification of some of the mechanisms that regulate interorganellar contacts and vesicle transport routes between peroxisomes and other organelles. However, important questions remain unanswered, particularly in the study of peroxisomal dynamics in unique cell types relevant to development and disease. The search for the molecular machinery and signals that govern both vesicle transport routes to peroxisomes and the establishment of functional contact sites with other organelles have just begun. It is clear that finding the answers to these questions will provide potentially groundbreaking insights into fundamental cell biology and into multiple disease pathologies.
